# Free hand hitting of stone-like objects in wild gorillas

**DOI:** 10.1038/s41598-022-15542-7

**Published:** 2022-07-15

**Authors:** Shelly Masi, Emmanuelle Pouydebat, Aurore San-Galli, Ellen Meulman, Thomas Breuer, Jonathan Reeves, Claudio Tennie

**Affiliations:** 1 Eco-Anthropologie (EA), Muséum National d’Histoire Naturelle, CNRS, Université Paris Cité, Musée de l’Homme 17 place du Trocadéro, 75016 Paris, France; 2 Department Adaptations du Vivant, UMR7179 MECADEV CNRS, Muséum National d’Histoire Naturelle, 55 rue Buffon, Paris, France; 3 Wildlife Conservation Society, 2300 Southern Boulevard, Bronx, NY 10460 USA; 4 Department for Early Prehistory and Quaternary Ecology, University of Tübingen, 72070 Tübingen, Germany; 5 Present Address: World Wide Fund for Nature – Germany, Reinhardstrasse 18, 10117 Berlin, Germany

**Keywords:** Anthropology, Archaeology, Behavioural ecology

## Abstract

The earliest stone tool types, sharp flakes knapped from stone cores, are assumed to have played a crucial role in human cognitive evolution. Flaked stone tools have been observed to be accidentally produced when wild monkeys use handheld stones as tools. Holding a stone core in hand and hitting it with another in the absence of flaking, *free hand hitting,* has been considered a requirement for producing sharp stone flakes by hitting stone on stone, *free hand percussion*. We report on five observations of *free hand hitting* behavior in two wild western gorillas, using stone-like objects (pieces of termite mound). Gorillas are therefore the second non-human lineage primate showing free-hand hitting behavior in the wild, and ours is the first report for free hand hitting behavior in wild apes. This study helps to shed light on the morphofunctional and cognitive requirements for the emergence of stone tool production as it shows that a prerequisite for free hand percussion (namely, free hand hitting) is part of the spontaneous behavioral repertoire of one of humans’ closest relatives (gorillas). However, the ability to combine free hand hitting with the force, precision, and accuracy needed to facilitate conchoidal fracture in free hand percussion may still have been a critical watershed for hominin evolution.

## Introduction

A large body of information on the evolution of human behavior and cognition is inferred from the archeological record. Neither tool making behavior nor its underlying cognition fossilizes, and so must be inferred from artefacts themselves^1^. Outside of a large number of stone tools from the Early Pleistocene, the only other interpretable evidence is bone tools, bone structures of the hominins themselves, and marks left on bone by tools^2–5^. Bone-based types of data are however extremely rare. Stone tools are available in much larger number, though even then the inferences that are drawn from stone tools remain limited. For example, the production techniques of the earliest stone tools in the hominin lineage (early knapping techniques) for which there is robust data (the Oldowan) have been variously argued to have been socially learned (e.g.^6^) or conversely to have been largely individually derived^7–9^. It is difficult to conclusively argue one way or another by reference to these stone tools alone, considering also additional problems of potential genetic and environmental influences on technique styles and/or equifinality and underdetermination of tools^10^.

Living primates provide an opportunity to gain additional insight into the factors that are required for the emergence of stone tool production. Recent work has shown that sharp stone flakes can come about unintentionally (albeit rarely) in wild primates^11^. Proffitt et al.^11^ report that flaked stone tools are accidentally produced when wild bearded capuchin monkeys (*Sapajus libidinosus*) hit naturally embedded stones with loose, handheld stones. Notably, the capuchins did not use the (sometimes) resulting flakes. They were rather interested in ingesting the stone dust that was produced. Similarly, wild chimpanzees (*Pan troglodytes*) sometimes accidentally produce stone flakes^12^ when missing a nut during nutcracking (here, they sometimes hit the anvil stone that is resting on the ground instead). However, in the case of the chimpanzees, the resulting flakes are not sharp, due to locally available stone material properties^12^. In both cases, the intention of capuchins and chimpanzees is not to produce sharp stone tools. Instead, capuchins seemingly intend to produce stone dust and chimpanzees intend to hit nuts on a stone anvil.

To date, the only primate species that has shown spontaneous, untutored evidence for not only intentionally *producing* sharp stone flakes (here, by hitting a fixed stone core of suitable material) but also *using* these flakes to cut their way towards rewards (in otherwise inaccessible puzzle boxes), have been captive tufted capuchins (*Sapajus apella*)^13^. Given that these tested capuchins were not only untutored, but also unenculturated, this shows that tufted capuchins have the natural sensory motor and cognitive abilities to make and use sharp stone flakes (at least when the core is fixed).

However, stone tool experts distinguish behavioral forms of stone tool production, the percussion styles, or knapping techniques. They differentiate the type of percussive styles by way of the relative localization and fixation of the hammerstone and the stone core (where the latter is defined as the stone from which flakes are taken). Free hand percussion is considered to be one of the primary strategies^6^. During free hand percussion, a hammerstone is held in one hand and this hand moves it to forcefully strike another stone, the core, held by and/or with a body part (e.g., the other hand). Free hand percussion is considered to be the primary mode of stone tool production of early hominins in the Oldowan^6,14^. Note however that the initial (currently debated;^15^) sharp stone tool production techniques perhaps lacked usage of the free hand percussion technique^16^.

We can describe free hand percussion consisting of two parts: (A) holding a stone core in one hand and/or on the body and *hitting* it with another (hammer) stone (*free hand hitting*), and (B) hitting stone on stone in the aforementioned way, but additionally being actually successful in producing sharp flakes in this way (*free hand percussion*). Note that B not only depends on the type of hammerstone (hard, and, ideally, rounded, as rounded stones tend not to break at impact) but also on the raw material of the hammer, and especially the core (able to produce sharp flakes from, ideally including some angles suitable for knapping). It also depends on where the core is hit, e.g. at exterior platform angles of 90 degrees or less^17^, *or* elsewhere if the goal is to “split up” a rounded cobble to create better knapping angles down the line. It also depends with how much force the action is executed (this force must be enough to initiate flake removal). Therefore, B may be claimed to be more encompassing, intentional and complex than A. Yet, A is a necessary precursor to B, and it is A (free hand hitting) that we will focus on here. For the first time we present evidence that a wild ape, the western gorilla (*Gorilla gorilla*), spontaneously shows *free hand hitting* behavior. During termite processing and feeding, two gorillas showed the specific behavioral form of free hand hitting, using two pieces of termite mound (i.e. stone-like objects).

Very few examples exist of stone handling behavior in unenculturated and untrained primates. Long-tailed macaques (*Macaca fascicularis*) use axe-shaped stones to smash the shells of rock oysters, detached gastropods, bivalves, and swimming crabs^18,19^. Japanese macaques (*Macaca fuscata*) also show so-called stone handling patterns, a behavior whose motivation is currently not well explained^20^. In this case, the stone handling repertoire includes also the behavioral form of free hand hitting. While there have been cases on free hand hitting behavior in captive apes, the tested ape subjects had received previous training and were also, at least to some degree, human-enculturated^21,22^. A recent study showed that unenculturated and untutored chimpanzees do not make or use flakes despite having been provided with loose stones and a motivation to make and use sharp stone tools^23^. Human training and human enculturation are factors that did not occur in the Oldowan^7^. Therefore, enculturated captive ape examples fail to provide ecologically relevant data and must be excluded for comparisons with Oldowan hominins (compare also^24^). We are unaware of any published data on ape free hand hitting behavior in wild and/or untrained/unenculturated (ecologically relevant) apes.

Among the wild apes, gorillas use tools the least^25^. Still, besides so-called pseudo tool use, such as throwing grass, sticks or branches to intimidate intruders observed in western gorillas^26^, both the western and eastern species of gorillas (*Gorilla gorilla* and *Gorilla beringei*, respectively) have also been observed to use tools, albeit rarely. For example, wild western gorillas have used detached branches, to test the depth of the water before crossing pools and create a self-made bridge from detached branches to cross floating aquatic vegetation^25^.

Wild mountain gorillas (*Gorilla beringei beringei)* use sticks to extract ants from their nest in chimpanzee-like ways^27^, and they also use a (detached) bamboo culm as a ladder to allow infants climbing up to reach food^28^. In addition, captive gorillas use tools with similar proficiency of other ape species (reviewed by^29–31^). This is likely due to the so-called captivity effect, an effect that does not seem to require human training or human enculturation to come about^32^. Thus, when properly motivated, gorillas` natural morphofunctional and cognitive capacities prove sufficient for them to have complex manipulative skills with high levels of sensorimotor abilities^33–37^*.*

**Figure 1:**
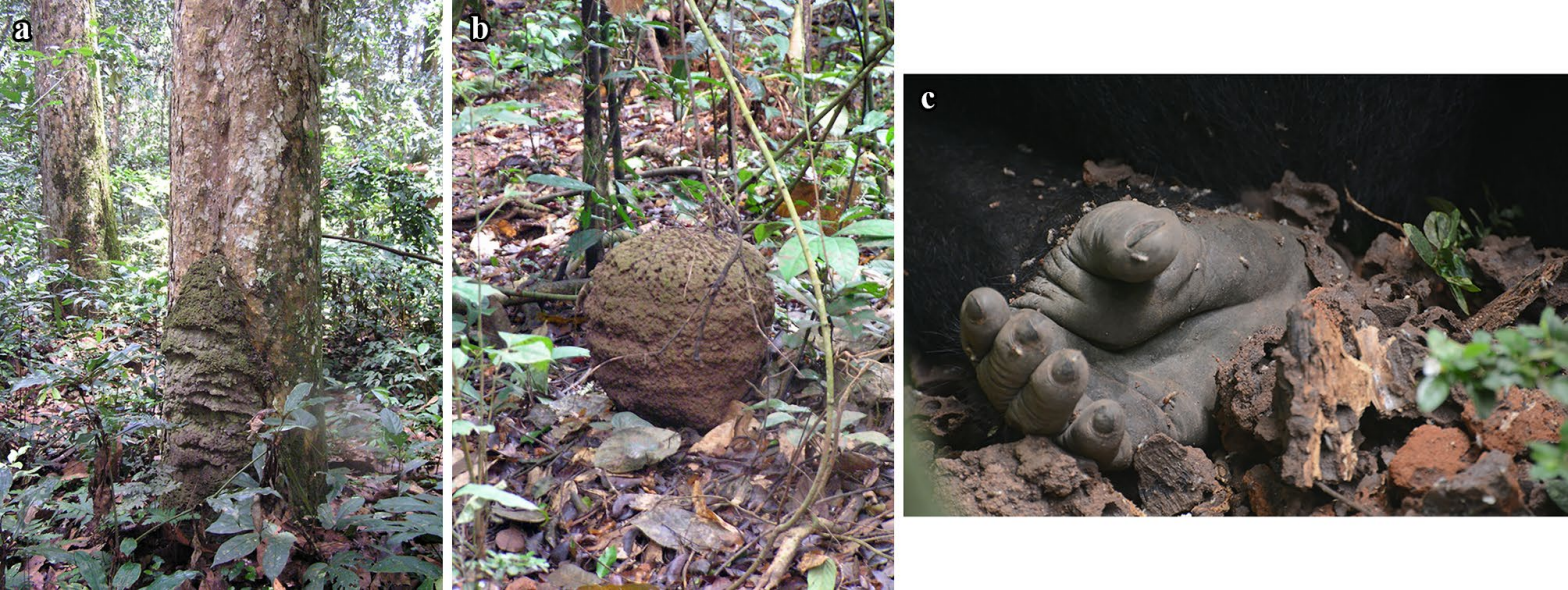
Tree (**a**) and ground (**b**) termite mound of *Cubitermes spp.*. External surface and cells with larvae of hand-size piece of termite mound of *Cubitermes* spp. broken by western gorillas (**c**). Photo credit: Shelly Masi.

The relative rarity of food that requires extractive foraging techniques has been considered the best candidate explanation for the low degree of foraging tools in wild gorillas^33,38^. However, wild western gorillas do feed by extractive foraging, for instance on termites and ants^28,39–41^. Termites are the most common insect prey for western gorillas, a highly nutritional resource particularly during the wet season^39,41–44^. When eating termites, gorillas appear to simply use their strength (transmitted via their arms) to break up mound pieces of hypogeic *Cubitermes* spp. from trees or the ground (Fig. 1a–c) while for the smaller chimpanzees is likely more efficient to use tools, i.e. to fish termites with sticks (epigeic nests of *Macrotermes* sp; western gorillas:^39^).

*Cubitermes* spp.’s mound consists of hard mineral-rich compacted solid soil sediments^45^, a stone-like material with properties somewhat similar to sand-stone. Moreover, 95–99% of the termite mound consists of clay, sand and minerals^46^. Given these characteristics, the pieces of termite mound that gorillas break off are hard and structurally similar to some natural stone in its properties. Henceforth, we will refer to pieces of termite mound as stone-like objects. This is not to say that this material (termite mound) has the suitable properties to produce sharp flakes when hit (it does not). This aspect (subsumed under knappability) is therefore missing from our data.

Here, we aimed to report a new behavioral observation on *free hand hitting behavior* using two stone-like objects (pieces of termite mound; Fig. 1a–c) in two immature wild western gorillas. Secondly, we aimed to compare the actions of this behavior with those of the usual termite feeding to assess whether the free hand hitting behavior could have been a variant of the usual feeding repertoire. In addition to the social context in which the behavior was displayed, we describe various stone-directed manipulative behaviors; classifying them according to the morphological aspects of dichotomous typologies of stone handling behavior (following^20^). We also investigated the proximate reasons that may have fostered the free hand hitting behavior by examining the behavior immediately preceding its occurrence. Since the ultimate goal of this study is to increase our understanding on the possible emergence of stone flake technology in hominins, we compare and discuss stone hitting in primates. In particular, we discuss the origins of stone tool production in hominins in general and the implications of our findings for the evolution of sharp stone flake production via free hand *percussion* in the human lineage in particular.

## Results

Using continuous focal animal sampling^47^ we daily recorded any feeding activity and videos of food processing in two groups of habituated western gorillas (number of individuals: Kingo = 13 and Buka = 10). The study groups have been observed simultaneously from March to June 2014 at the Mondika Research Center in the Djeke Triangle, in the Republic of Congo. The behavior we report here was observed and captured on video during full-day focal observations (Kingo: *N*_days_ = 33, *N*_hours_ = 213. Buka: *N*_days_ = 21, *N*_hours_ = 145). *Free hand hitting* behavior was coded whenever (1) a gorilla held one piece of termite mound in one hand and another piece of mound in the other hand, as long as (2) the gorilla hit one of the mound pieces against the other mound piece. In addition, we recorded the hand used (right or left) to pick up, hold, pound and ingest mounds/termites.

**Figure 2:**
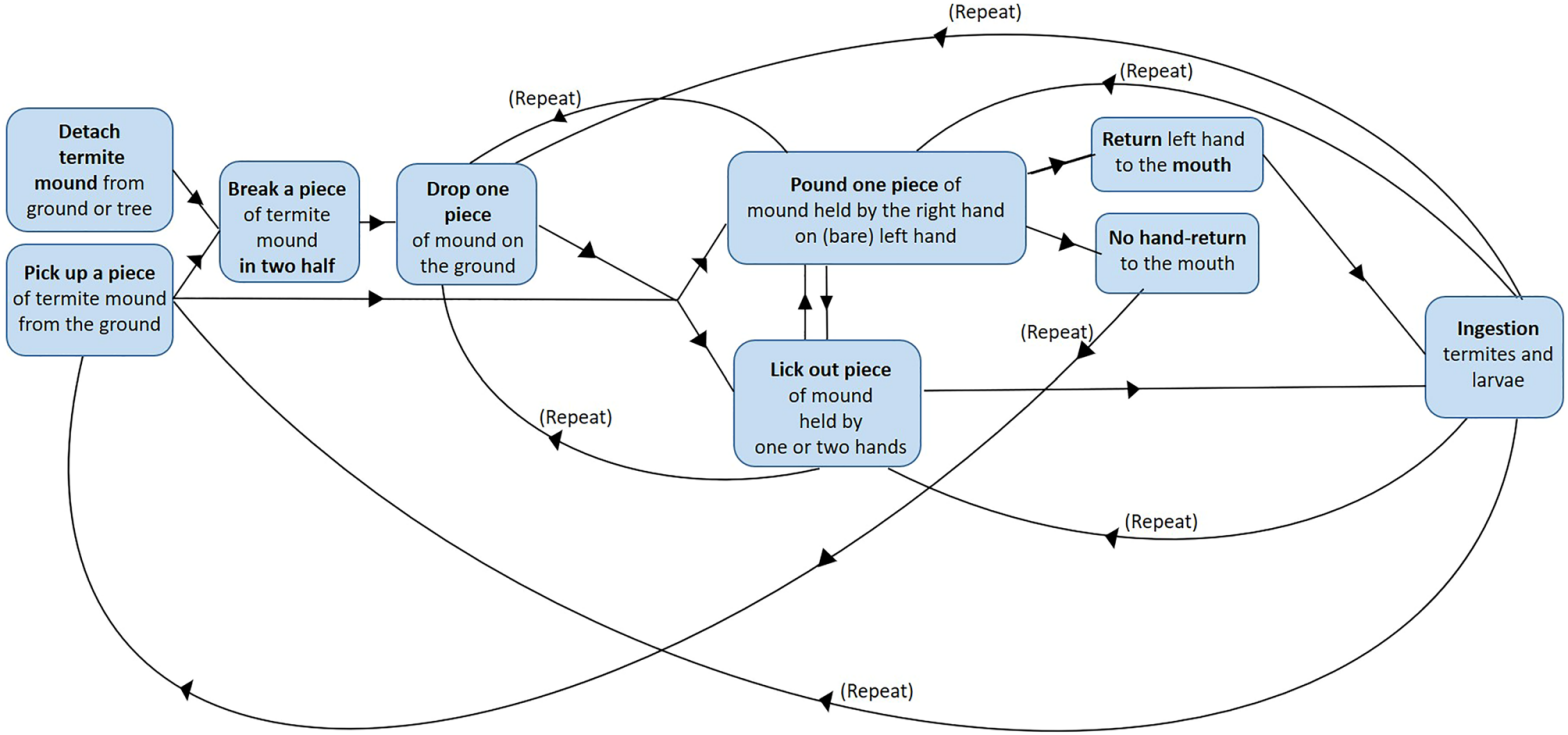
Behavioral steps of the usual pounding technique to extract termite larvae of *Cubitermes* spp. by wild western gorillas.

**Figure 3:**
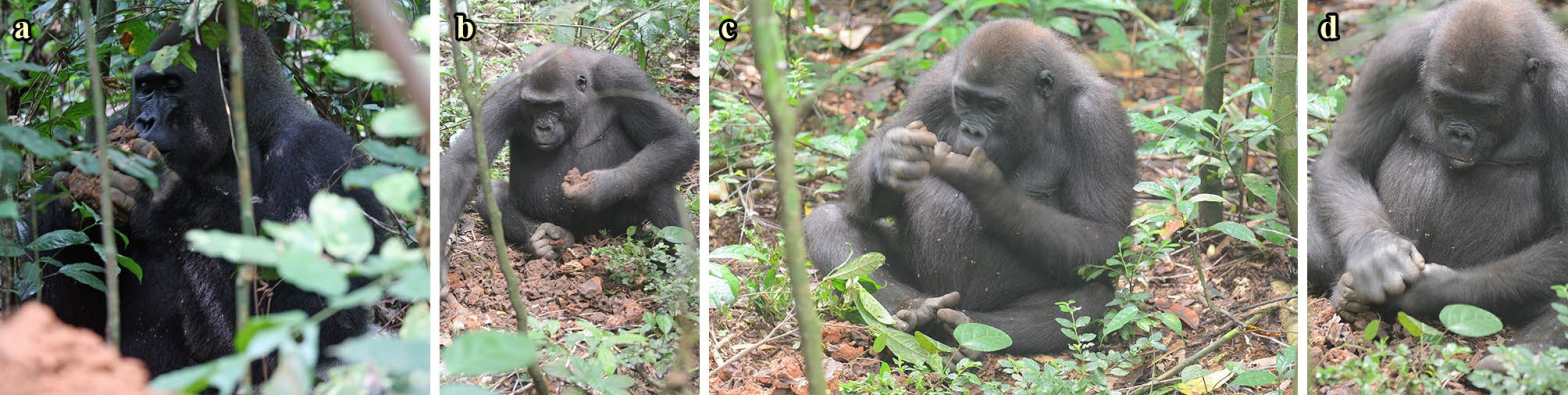
Termite feeding licking technique (**a**). Gorilla picking from the ground a piece of termite mound (**b**), bringing termites to the mouth after pounding (**c**) and bimanually breaking further in half the mound piece. Photo credit: Shelly Masi.

**Figure 4:**
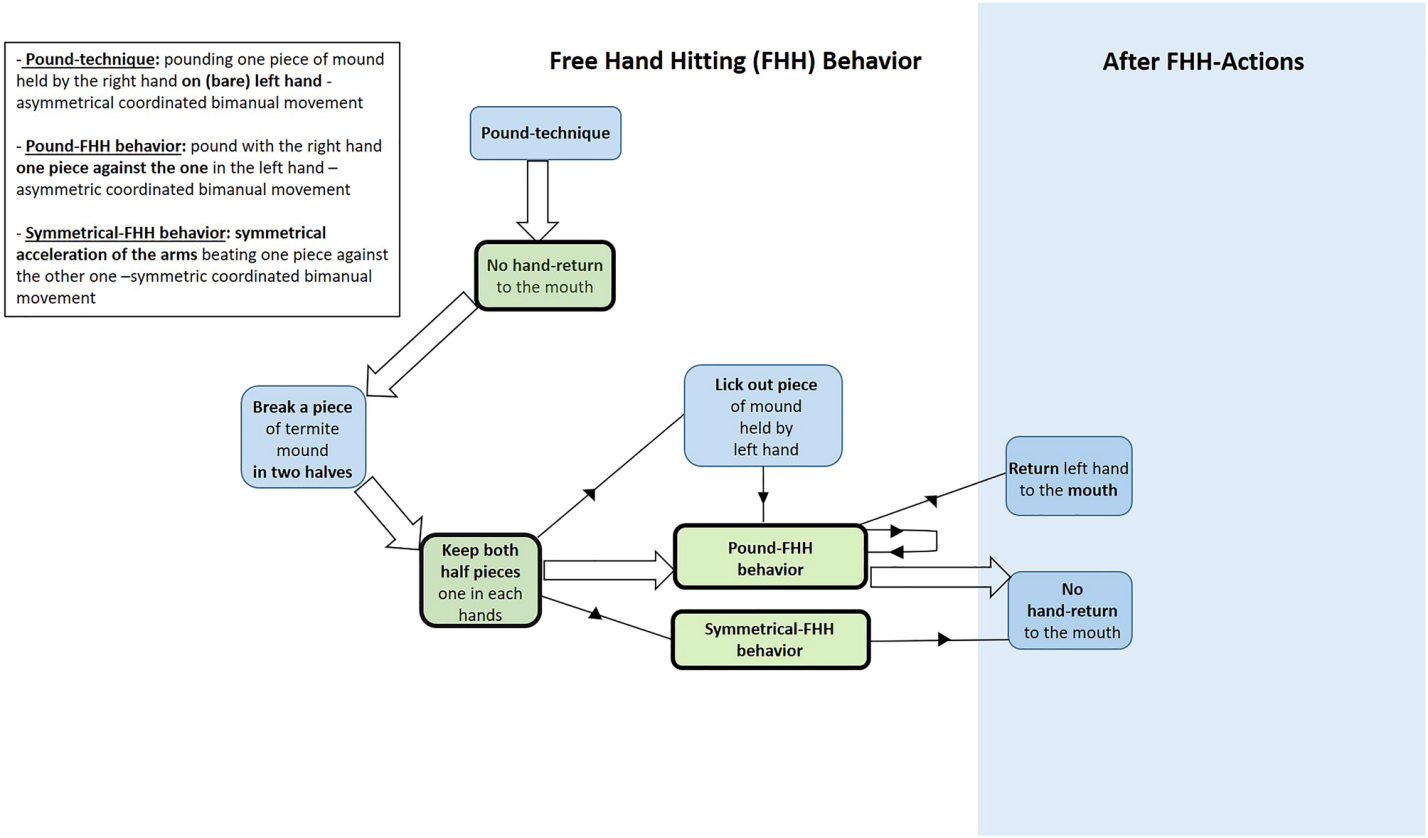
Simplified action style analysis of the behavioral variants of the pounding technique, Free Hand Hitting (FHH) behavior, displayed by two infants of western gorillas while feeding *Cubitermes* spp. termites. Large arrows show common steps to all observed FHH. Small arrows show actions present in at least one FHH observed. In green are highlighted the novel behavioral actions in comparison to the usual termite processing. For a more detailed action style analysis of each FHH behavior see the Fig. 1 ESM.

All study individuals fed on *Cubitermes* spp. termites by extracting them from mounds (via detached pieces; Fig. 1a–c). In one of the two study groups (Kingo group) we observed two gorillas, a four-year-old infant (male, Etefi) and his younger half sibling (male, Ika) deviating from the usual “pounding technique” (Figs. 2 and 3a–d). Both individuals instead engaged in free hand hitting behavior using two pieces of mound (Fig. 4 and Supplementary Video 1). This happened once in Etefi’s case and four times in Ika’s case (Fig. 1 ESM). Both individuals broke a termite mound piece in two halves (roughly of the size of their hand) and rather than dropping one of the two on the ground (as in the gorillas’ usual processing), they kept both mound pieces; one in each hand. They then used bimanual coordinated actions to pound these stones *against another* (Fig. 4). Both individuals thus fulfilled our coding criterion for *free hand hitting* behavior. Coordinated symmetric and asymmetric techniques were shown (Fig. 4 and Supplementary Video 1). Both infants showed *free hand hitting* behaviors with coordinated asymmetric actions (Etefi once and Ika three times; Fig. 4 and Fig. 1 ESM). Ika additionally showed coordinated symmetric free hand hitting behavior (once; Fig. 1 ESM). During the five events of *free hand hitting* behavior, none of the termite mound pieces broke into smaller pieces and thus no flake-like pieces were produced (Supplementary Video 1). The specific manipulative behaviors of these five cases are summarized in Fig. 4. The details per action and individual are described in ESM and summarized in Fig. 1 ESM.

For comparison, adult and immature western gorillas generally use two different feeding techniques to extract termites (^39,40^; see schematization in Fig. 2, Supplementary Video 2 and Fig. 3a–d). After breaking a piece of termite mound in two halves using both hands (Fig. 3a-d; Supplementary Video 2), gorillas generally extract termites either by licking termites exposed on the surface of the mound pieces held in one or two hands (unimanual or bimanual *licking technique*) or by pounding pieces of termite mound held in one (the right) hand on the other hand (their left hand; the *pounding technique*: Supplementary Video 2 and Fig. 3a–d). In the latter technique, termites fall out from the piece of mound (from the external openings of the mound cells; Fig. 1c) onto the gorilla hand and the gorillas then ingest fallen termites by bringing the hand containing the dropped termites to the mouth (Figs. 2, 3a–d and Supplementary Video 2). After some time, the gorillas drop the termite mound piece, perhaps when it is exhausted of termites. They may also break it again, perhaps to re-initiate efficient foraging. The bimanual actions used by the two gorillas during their free hand hitting movements resemble the usual pounding technique, but with a major difference. In free hand hitting, there is no empty hand, as one piece of termite mound is held in each hand.

We cannot exclude that the two gorillas who performed free hand hitting behavior might have done so also outside of our observation of them (e.g. in times in which we observed other group members). If they did, they might have observed each other performing free hand hitting behaviour. As for the observations we report here for free hand hitting behaviour in gorillas, only in one feeding session could it have been possible that one individual observed the other (see below).

During the feeding session where Ika was observed performing free hand hitting behavior (on 29th May 2014), only one other individual was visible. At the end of the tape a large individual (not fully visible) walked nearby Ika who left the site two seconds later (maybe this was Ika’s mother, but it certainly was not Etefi; Supplementary Video 1). That is, Etefi did not observe Ika’s free hand hitting behaviour in this feeding session.

One week before this observation (on 12th May 2014), during the feeding session in which Etefi *was* observed to perform free hand hitting behavior, Ika could have observed Etefi (from a distance). During this session, only two other individuals were present at close range from Etefi: his mother and his older full brother, both feeding termites within 0.5 m from Etefi (Supplementary Video 3 ESM). However, during the same feeding session, three other individuals can be seen on our video recordings in Etefi’s proximity (approximately 4 m away from Etefi): an adult female feeding termites, a juvenile moving, feeding and monitoring the environment, and two younger infants moving with the juvenile and observing the adult female feeding (Supplementary Video 3 ESM). Being these individuals far in the back of the video, their identification is difficult. During the study period only two infants of similar size to the ones seen in the video were present in the Kingo group. Thus, one of the two infants we saw on video was certainly Ika (who could have possibly observed the free hand hitting behaviour in this instance).

## Discussion

We reported free hand hitting behavior, using stone-like objects (termite mound pieces), in a wild ape, the western gorilla. Across several instances, two young gorillas hit two hand-held pieces of *Cubitermes* termite mounds against another. Because of the underlying behavioral form (hitting two hand held objects against each other) and because termite pieces are hard mineral-rich compacted soil (not real stones, but fairly hard objects), we interpret these behaviors as the first observation of free hand hitting behavior in wild apes.

Free hand *hitting* behavior is of interest, as it is a prerequisite behavioral form for free hand *percussion*, in which sharp stone flakes become detached from suitable stone. Even though we acknowledge a low number of observations on a low number of individuals (two), these rare observations per se nevertheless show that gorillas have both the morphofunctional manipulation abilities, the sensorimotor skill and the environmental stimuli required to perform free hand hitting behavior. Therefore, wild gorillas can be considered the second non-human lineage primate to show spontaneous, untutored free hand hitting behavior in the absence of human enculturation (besides Japanese macaques;^20^).

These observations of free hand hitting behavior in the wild suggest that the wild foraging context might have triggered this behavior not only in gorillas, but perhaps also in the last common ancestors of gorillas and humans. Note that we do not wish to claim that the free hand hitting of the two gorillas, using termite mound pieces, could have led to the production of sharp stone tools, such as flakes. We merely highlight that these actions closely resemble free hand hitting behavior involved in a major flake production technique (free hand percussion). This helps to shed light on the emergence of stone flake technology, as the underlying basic actions seem within the range of ape behaviour today (at least within the range of gorilla behaviour). Unusual with regard to the usual termite feeding techniques, the two gorillas we observed to use free hand hitting actions kept one piece of mound in each hand and then, they impacted one mound with the other. While this behaviour was in other ways similar to the usual technique, as here gorillas pound a single termite mound piece held by one hand into the empty palm of another hand (usual pounding technique; Supplementary Video 2 and Fig. 3a–d), the adding of another piece of mound in the otherwise empty hand rendered the action free hand hitting behaviour. In a similar way, knapping to produce flakes requires a coordinated bimanual action. In addition, in the majority of free hand hitting behaviors of the two gorillas (*N* = 4) the action was performed by one leading hand (e.g.^48,49^) in an asymmetric way. This, too, happens in free hand percussion^50^. Note however that one of the two gorillas we observed to perform free hand hitting behaviour (Ika), in one instance changed the asymmetric free hand hitting action to a more *symmetrical* acceleration of the arms (possibly attempting to increase efficiency, though this cannot be ascertained).

The coordinated asymmetrical bimanual free hand hitting pattern may have been a variant of the usual (likewise asymmetrical) pounding technique of gorilla termite feeding. Both require manual role differentiation and some degree of functional cerebral asymmetry. In addition, the required learning time and the late emergence during the development (c.a. 2-year-old age;^51^, Masi personal observation) of the coordinated asymmetrical termite pounding technique is consistent with an assumption of a relatively high difficulty of the manipulations performed during free hand hitting behavior.

Given these similarities between the free hand hitting and the usual gorilla pounding technique, it seems at least plausible that the gorillas performed these actions with the intent to open the termite mound piece by hitting it with another piece of mound. Alternatively, the gorillas may have intended to directly make more termites come out from the mound pieces by using a harder substrate than their hand (like in proto-tool use, i.e. the transformation of an object by its application to a stable, usually hard or resistant substrate;^52–55^).

Furthermore, the action style analysis of the younger of the two infants, Ika, highlights that the action preceding all his four free hand hitting behavioral instances was a lack of ingestion of termites after using the usual pounding technique. Particularly, the key common action for all five free hand hitting events (across both individuals) was the decision to retain two pieces of termite mound, one in each hand, after breaking a larger termite mound piece in two. Exploratory attempts to test for alternative termite extractions, after a series of unsuccessful termite harvesting efforts (i.e. Ika, Fig. 1 ESM) may have triggered free hand hitting behavior. However, the free hand hitting behavior was likely inefficient for both individuals: The termite mound fragments never broke and no ingestion action of termites followed, except for one case (Etefi).

Primate infants are generally less efficient, and can lack of the full coordination and precision needed for the customized processing movements of adults, particularly in great apes^56^. However, both infants evidence that they had fully acquired pounding techniques before and after displaying the free hand hitting behavior. Likely, an insufficient speed and acceleration may be responsible for the inefficiency of the infants’ free hand hitting actions since the movement itself (flexion extension, pronation) seemed to be appropriate. They may have simply attempted to find an alternative action on a similar same behavioral pathway to compensate for foraging inefficiency, not least since termites may be important for the high energetic growth demand of immatures like them^57,58^.

Our study underlines that the development of tool-use and foraging competence in primates (including humans) are linked to skilled actions acquired through the routine generation of species-typical exploratory actions, or play behavior coupled with learning about the cost/benefits of each action^20,59^. An alternative explanation to free hand hitting being perhaps a foraging variant, is that this behaviour resulted from *playing* with the mound fragments. However, the infants did not appear to be in a playful attitude but rather concentrated on feeding (Masi, personal observation). And so, even though this alternative “play hypothesis” cannot be fully excluded, this and the similarity of the free hand hitting actions with the foraging ones may render this explanation less likely.

In western gorillas, social learning among immatures is possible since they observe often their peers in comparison to adults^60^. Some social learning also cannot be fully excluded here since the two individuals belonged to the same study group. Even though the two infants were not facing each other at close distance, Ika was at least nearby when Etefi engaged in free hand hitting behavior (see ESM Supplementary Video 3). However, at the very least, logically, *one* of the infants would have been required to innovate free hand hitting behavior as this behaviour is not usually performed in this group (or any group we know of; Masi, long term data).

Even though we acknowledge that free hand hitting behavior is relatively rare (observed five times in only one of the study groups, over 299 total minutes of videotaped termite feeding for both groups), it directly adds to the very rare records of tool use observed in wild gorillas^25–27^. Though of course, the attempt of tool use here, via free hand hitting, was unsuccessful.

Free hand hitting behavior in gorillas is another example of (intentional) attempt for stone breakage by a non-human primate, even though in our case this did not (and could not, due to material properties) produce sharp stone objects like in wild^11^ or captive capuchins^13^. However, to date, western gorillas are now the only wild primates that display free hand hitting actions whereby they may bimanually crush stone-like surface by way of hitting one (stone-like) object with another. They did this perhaps with the intention of breaking the pieces, though not to produce sharp objects but to extract termites.

In contrast, capuchin monkeys seem to use stone tools in more varied activities (pounding foods, digging and in sexual displays;^61–63^) than gorillas, who used stone-liked objects in the context of termite foraging only. However, in those cases in which wild capuchins made flake-like objects, they display a clear asymmetric bimanual pounding action while striking the active stone against the passive hammer or substrate^11^. Japanese macaques^20^ show coordinated symmetric actions while pounding stone. The gorillas we observed seemed more flexible and used both symmetrical and asymmetrical actions to pound stone-like objects. Like for wild capuchins during dust production, and like some stone handling actions of macaques^11,20^, the gorilla bimanual coordinated precision and power, in combination with percussive actions, are reminiscent of percussive actions in early hominins (cf.^64–67^). Powerful high-speed actions combined with elastic energy has been suggested as critical feature for human evolution^68–70^. Perhaps, even though the observed free hand hitting by the two immature gorillas may have not been powerful or elastic enough to break the termite mounds, it reflects an important, logically necessary, first step in being able to perform such action (see Introduction).

As discussed above, there are several requirements for the production of flakes via chonchoidal fracture. It is clear from this study and others that the basic manual actions required for such flake production exist across multiple primate taxa. In fact, some of these actions do result in products that are similar to or indistinguishable from flakes (in capuchins see^11^). However, given that no wild primate species make use of these flakes^11^, it can be argued that primates do not intend to initiate conchoidal fracture (unlike *captive* capuchins^13^), and that conchoidal fracture, though infrequent, can be somewhat reliably achieved in the absence of intention. Thus, the main ultimate question may be not whether a species possess the biomechanical capabilities to hit two stones together, but rather whether they can be combined in such a way to systematically produce flakes and use them. In this sense, it may be more useful to discuss how these actions are combined as opposed to whether or not they exist in the wild.

To this end, the ability to combine these various actions may have been a critical threshold in hominin evolution. The ability to *reliably* initiate conchoidal fracture via free hand percussion and to then use the sharp edges resulting to extract otherwise inaccessible or less accessible resources may be the crucial difference between our lineage and non-human primates. By showing that a logical prerequisite for free hand percussion, free hand hitting, can already be part of the natural developing behavioral repertoire of one of our closest relatives, gorillas, our study helps to shed light on the likely range of requirements and potential sources for the emergence of such stone tool production and use during human evolution. Particularly, even though throughout human evolution tool use behavior may have increased alongside a gradual evolution of human-like morphology^71^, our current knowledge on extant non-human primates underlines that species with very different skeletal morphologies and locomotor patterns (e.g. arboreal capuchins and semi-terrestrial western gorillas) have the biomechanical and morphofunctional capacity to knock two rocks together.

## Methods

### Study site and study groups

Two groups of habituated western gorillas (Kingo and Buka group) with overlapping home ranges were observed at the Mondika Research Center in the Djeke Triangle, managed by the Wildlife Conservation Society in the northern part of the Republic of Congo at the boundaries with the Central African Republic. The study site is composed of semi-deciduous rain forest of low altitude (< 400 m) and it receives an average of 1600–1800 mm rainfall per year^72^. During the study period, the two gorilla groups did not change group compositions (number of individuals: Kingo = 13 and Buka = 10) and consisted of one silverback (in each group), five and two adult females (group Kingo and Buka, respectively), one nulliparous female (group Kingo), one young silverback (group Buka), one and two blackbacks (group Kingo and Buka, respectively), one subadult in each group, two and one juvenile (group Kingo and Buka, respectively) and two and one infants (group Kingo and Buka, respectively; age-sex classes according to:^73^). All research adhered to the protocols and legal requirements for Republic of Congo. All observations were performed in accordance with it.

### Data collection

Data were collected each day simultaneously on both groups from March to June 2014 by SM, AS and EM. Using continuous sampling we recorded any feeding activity including termite feeding by focal individuals^47^ during full-day observations (group Kingo: *N*_days_ = 33, *N*_hours_ = 213; group Buka: *N*_days_ = 21, *N*_hours_ = 145). We rotated the focal individuals each half-day to ensure that within each group every individual was sampled at least twice per month and that morning and afternoon observation were balanced. The total amount of minutes of termite feeding behavior tape recorded for group Kingo and Buka was 174 and 125 min, respectively: two silverbacks (38 min), five adult females (111 min; two females were not recorded), one young silverback (8 min), three blackbacks (71 min), three subadults (19 min; including the nulliparous female), three juveniles (23 min), three infants (31 min).

In addition to focal continuous sampling data, videos of food processing (including termite feeding) were daily collected and were coded by AS and SM with the software ELAN3.eaf. The behavior we reported here was observed and captured on videos during focal-follows. During termite feeding we recorded whether the focal individual was observed by conspecifics, and if so, the identity of the observer(s) and of all other visible individuals in the video estimated ≤ or > 5 m from the focal individual.

### Manipulative skills quantification

Detailed information on the actions (behavioral forms) made by the focal individual before and during the termite ingestion was recorded. *Free hand hitting* behavior was coded whenever (1) a gorilla held one piece of termite mound in one hand and one piece of termite mound in the other hand and (2) when they then hit one piece of termite mound against the other piece of termite mound. In addition, we recorded the hand used (right or left) to pick up, hold, pound and ingest termites/mounds. For each video clip of termite feeding we defined a feeding session as the period of time in which the focal individual continuously fed (pausing or changing activity for < 30 s) on the same termite mound (which could consist of feeding on multiple pieces of termite mound). We assumed that termite feeding occurred when the individual made the ingestion action to bring a piece of termite mound or a hand with termites on it, to the mouth with one or both arms. An unsuccessful extractive-action or no ingestion was defined when the individual did not perform the ingestion action to bring the mound piece/hand to the mouth (thus when no contact between the mouth and the piece or the hand holding termites occurred).

In bimanual actions, the active hand was defined as the dominant or leading hand considered as producing the action itself (e.g.^48,49^). To describe the form of bimanual free hand hitting behavior, we used the criteria of coordination (uncoordinated vs. coordinated) and symmetry (symmetrical vs. asymmetrical) following Leca et al.^20^. Uncoordinated bimanual manipulation occurred when both hands performed actions independently of each other in space and/or time, whereas coordinated bimanual manipulation occurred when both hands performed actions working together to achieve a unitary goal, e.g. hitting a piece of termite mound on the other palm hand or on another piece^20,74,75^. Following Leca et al.^20^ symmetrical bimanual manipulation was coded when both hands performed the same action (simultaneously or alternatively), whereas asymmetrical bimanual manipulation was coded when both hands performed different actions simultaneously^75^.

## Supplementary Information

Supplementary Video 1.

Supplementary Video 2.

Supplementary Video 3.

Supplementary Information 1.
