# Publisher Correction: Free hand hitting of stone-like objects in wild gorillas

**DOI:** 10.1038/s41598-022-17577-2

**Published:** 2022-08-24

**Authors:** Shelly Masi, Emmanuelle Pouydebat, Aurore San‑Galli, Ellen Meulman, Thomas Breuer, Jonathan Reeves, Claudio Tennie

**Affiliations:** 1 Eco‑Anthropologie (EA), Muséum National d’Histoire Naturelle, CNRS, Université Paris Cité, Musée de l’Homme 17 place du Trocadéro, 75016 Paris, France; 2 Department Adaptations du Vivant, UMR7179 MECADEV CNRS, Muséum National d’Histoire Naturelle, 55 rue Buffon, Paris, France; 3 Wildlife Conservation Society, 2300 Southern Boulevard, Bronx, NY 10460 USA; 4 Department for Early Prehistory and Quaternary Ecology, University of Tübingen, 72070 Tübingen, Germany; 5 Present Address: World Wide Fund for Nature-Germany, Reinhardstrasse 18, 10117 Berlin, Germany

Correction to: *Scientific Reports* 10.1038/s41598-022-15542-7, published online 15 July 2022

In the original version of this Article Shelly Masi was incorrectly affiliated with ‘World Wide Fund for Nature - Germany, Reinhardstrasse 18, 10117 Berlin, Germany.’ The correct affiliation is listed below.

Eco‑Anthropologie (EA), Muséum National d’Histoire Naturelle, CNRS, Université Paris Cité, Musée de l’Homme 17 place du Trocadéro, 75016 Paris, France

Additionally, the order of the accompanying Supplementary Video 1, Supplementary Video 2 and Supplementary Video 3 was incorrectly shown as Supplementary Video 2, Supplementary Video 3 and Supplementary Video 1.

The original Article and accompanying Supplementary Information files have been corrected.

