# Supplementary material for: Free hand hitting of stone-like objects in wild gorillas
Source: Sci Rep. 2022 Jul 15;12:11981. doi: 10.1038/s41598-022-15542-7 (PMC9287431; doi:10.1038/s41598-022-15542-7)

Free hand hitting of stone-like objects in wild gorillas

Shelly Masi[1*], Emmanuelle Pouydebat[2], Aurore San-Galli[1], Ellen Meulman[1], Thomas Breuer[3], Jonathan Reeves[4], and Claudio Tennie[4]

[1] Eco-anthropologie (EA), Muséum national d'Histoire naturelle, CNRS, Université de Paris, Musée de l'Homme 17 place du Trocadéro 75016 Paris, France

[2] UMR7179 MECADEV CNRS/MNHN, Department Adaptations du Vivant, 55 rue Buffon, Paris, France

[3] World Wide Fund for Nature – Germany; Reinhardstrasse 18; 10117 Berlin, Germany http://orcid.org/0000-0002-8387-5712

[4] Department for early prehistory and quaternary ecology, University of Tübingen, 72070 Tübingen, Germany

*shelly.masi@mnhn.fr

Supplementary Information

Detailed description of free hand hitting actions

Older Infant (Etefi)

During 25 min of termite feeding video records of Etefi (a four-year-old infant of group Kingo) he was observed performing *free hand hitting* behavior at least once during the same feeding session (whole session lasted 365sec and happened on 12th May 2014). In this feeding session, Etefi first licked several pieces of termite mound while these were still on the ground (ca 18sec) and then detached a small piece of the mound (smaller than his hand) attached to a nearby

tree. Then he began feeding on termites with the pounding technique (Fig. 2, 3) thus by pounding twice the mound piece that he had previously detached from the termite mound tree. Etefi used this pounding technique twice, successfully. He then dropped the mound piece and picked up another piece from the ground, probably bimanually breaking it in two pieces (not visible but there is a clear movement of the pectoral muscle) as usually gorillas do to break a mound piece and acceed to the new inner cells. Then, Etefi kept one large piece of mound in the right hand and he used the licking technique on it for about 10 sec. He then used the pounding technique with this piece, by pounding it on his left hand (three times). Only once, on the third pounding action, did he ingest termites (bringing the left hand to the mouth). Then Etefi bimanually broke the mound piece into two. He repeated this several times, in each case keeping only one of the resulting two pieces (always in his right hand) and dropping the other piece. This was followed by two instances of the pounding technique. Only the first one of these applications of the pounding technique was followed by an ingestion action. He then broke the remaining piece in half, and then dropped one of the pieces. He then broke the remaining piece again (though note: visibility at this point in the video is not ideal), repeating the aforementioned process. He then applied the pounding technique to the remaining mound piece once, followed by ingestion. He then repeated pounding and ingestion once. Then he broke the piece of mound again, dropping one piece. Then, he used the bimanual breaking action to break the piece again into two. This time Etefi kept both pieces of mounds in his hands (one in each hand). He then looked attentively at the pieces, for a second or so. It was then that he hit one mound piece on the other – showing *free hand hitting* behavior - using coordinated and asymmetric movements. Etefi hit the mound piece held in his left hand with the mound piece held in his right hand – twice (i.e. two instances of *free hand hitting*

behavior; Video 1 and ESM Fig. 1). His free hand hitting action was coordinated and asymmetric action, similar to the pounding technique. He then used an ingestion action once.

What followed was more termite ingestion, and one potential case of *free hand hitting* behavior (see below). After the above, Etefi hammered the piece held in his left hand with the piece held in his right hand twice. He then used an ingestion action once. Etefi then dropped the piece held in his right hand. This was followed by him breaking the mound piece previously held in his left (using both hands). Etefi proceeded to use the licking technique (twice) on one of the newly produced pieces (the one held in his left hand), while keeping the other newly produced piece in his right hand. Next, Etefi hit the mound piece held by his right hand onto the left hand. However, the visibility on the video is not sufficient to assess if the left hand still held the piece of mound at the end of this action. That is, this might have (possibly been an additional instances of free hand hitting. To be conservative, we did not include this unclear instance in the action analysis. No ingestion action followed. Etefi then dropped the piece(s) of mound to the ground. Afterwards the session continued (for 240 sec), but no further cases of *free hand hitting* behavior (not even potential cases) were observed.

Younger infant (Ika)

We observed a second set of *free hand hitting* behavior - on 29th May 2014. During 4 min of video tape for Ika feeding termites during the study period, this three-year-old male infant of the Kingo group was observed four times performing *free hand hitting* behavior, similar to those observed 17 days before by his older half sibling, Etefi. The termite feeding session with *free hand hitting* behavior (93 sec) started with Ika feeding on termites using the pounding technique (twice). This was unsuccessful (no ingestion action followed). Using both hands, he then manually broke the single piece of termite mound that he held at the time into two

pieces. At this point Ika retained both mound pieces, one in each hand. He licked the piece held by the left hand. Then, using an asymmetric and coordinated action he accelerated both the mound piece held by her right hand and that held by her left hand, one against the other, hitting the two pieces one against the other one showing *free hand hitting* (FHH) behavior (Ika FHH-action 2, Video 1 and ESM Fig. 1). No ingestion action occurred. Despite a slight acceleration of the left limb, in addition to that of the right limb as it occurs in the pounding tecnique, we classified the overall main action as asymmetric (made mostly by the movement of the right limb). Indeed, as during the pounding technique the main action was performed with the right elbow and hand (both performing flexions and pronations) that grasps the object and hits it against the left hand that in this case allowed hitting the mound piece held in the right hand with that in the left one. The movements of the left upper limb were simpler: while the left wrist moved but only a little (slight flexion-extension), the left elbow clearly moved (flexion). No ingestion action occurred. Then, Ika dropped both pieces he had in her hands on the ground, and picked up a large piece of termite mound from the ground. Ika then attempted again the pounding technique with this piece, but this was unsuccessful (no ingestion action followed). He then bimanually broke the piece of mound he held, resulting in a largish and a smallish piece of mound. Ika retained the larger part in her right hand and then he pounded the larger mound piece with the left hand that was holding the smaller part – showing free hand hitting behavior (asymmetric; Ika FHH-action 2, Video 1 and ESM Fig. 1). Just immediately after that both mound pieces did touch each other Ika dropped the smaller part from her left hand. No ingestion action followed. Ika bimanually broke again the remaining piece which resulted in two roughly equally sized mound pieces. He then looked attentively to both newly produced pieces for about a second, each held by one hand, and then he dropped the slightly smaller one, held by her left hand, on the ground. Ika used the pounding technique with the

remaining piece, and he then used an ingestion action (briefly). He then used the pounding technique again with the same piece, but no ingestion action followed. Then, Ika licked her right wrist hair (possibly some termites there) while still holding the mound piece in her right hand. Then he broke the mound piece he held, using both hands, resulting in two pieces of similar size, one piece each per hand. Ika then proceeded to hit these two pieces against each other – i.e. showing another case of free hand hitting behavior. This time the action free hand hitting behaviour was based on coordinated but roughly symmetric movement of the arms, accelerating both pieces toward each other in similar ways. We therefore classified this as symmetrical free hand hitting behavior (Ika FHH-action 3; Video 1 and Fig. 1 ESM). No ingestion action followed and Ika immediately dropped the piece he held in her right hand. He then moved the remaining piece from the left hand to the right one and used with it the pounding technique. Ika then paused (ca 2 sec) to look at the left hand, likely to visually inspect if termites had fallen off. No ingestion action followed. Then, he repeated the pounding technique twice. In both cases, no ingestion action followed. Ika then picked another piece of mound with her left hand from the ground, while he dropped the mound piece he held in her right hand. He bimanually broke the new piece in two parts, dropped one of the then two pieces and then continued to use the pounding technique for five times each followed either by a brief lick of the right wrist hair (twice) or by ingestion action (twice), except for the last iteration for which no ingestion followed. Ika then briefly tried to manually break the piece with both hands, unsuccessfully, and then he used the pounding technique again, but no ingestion action followed. Ika then dropped the piece, picked up another piece from the ground and used the pounding technique, but again, no ingestion action followed. He then picked another piece from the ground (with her left hand) while simultaneously dropping the one he held with the right hand. Ika then moved the piece held with the left hand into the

right hand and used the pounding technique. No ingestion action followed. Using two hands, Ika then broke the piece in unequally sized pieces, looked at them briefly and then he dropped both pieces. He then picked up another piece from the ground, with the left hand. Ika then used the pounding technique with this new (small) piece and used ingestion actions afterwards. He then tried briefly to break this piece (with both hands) but he failed thus he repeated the pounding technique. Again, no ingestion action followed and then Ika dropped the piece to pick up another (larger) piece. He then successfully broke this new piece (with both hands) in two: a large piece, which Ika held in her right hand, and a smaller piece, which he then held in her left hand. He paused (ca 2 sec) while looking at these two pieces. Then, Ika pounded the right hand with the larger piece on the small one held in the left hand – another case of free hand hitting behavior (Ika FHH-action 4, Video 1 and ESM Fig. 1). This time the movement was more asymmetric than the previous free hand hitting with the leading hand (the right one) clearly pounding its mound piece on the piece held by the left hand. Similarly to Ika FHH-action 2, here, even though both hands were acting, the right limb moved more and acted in a different way (pronation) from the action of the left limb (for which pronation was not present). Ika then looked at his hands (ca 3sec) and then dropped both pieces on the ground. He then left the site.

Fig. 1 ESM. Detailed action style analysis of the behavioral variants of the pounding technique, Free-Hand Hitting (FHH) behavior, displayed by the two infants of western gorillas while feeding *Cubitermes* sp. termites. In contrast to Figure 1, here each FHH episode has a distinct trait line and it is described from the last step of the usual pathway of termite feeding (detailed in Fig. 2) till the two following actions after each FHH behavior. Empty small arrows indicate

the starting point for each behavioral pathway description. In green are highlighted the novel behavioral actions in comparison to the usual termite processing.

Figure 1 ESM

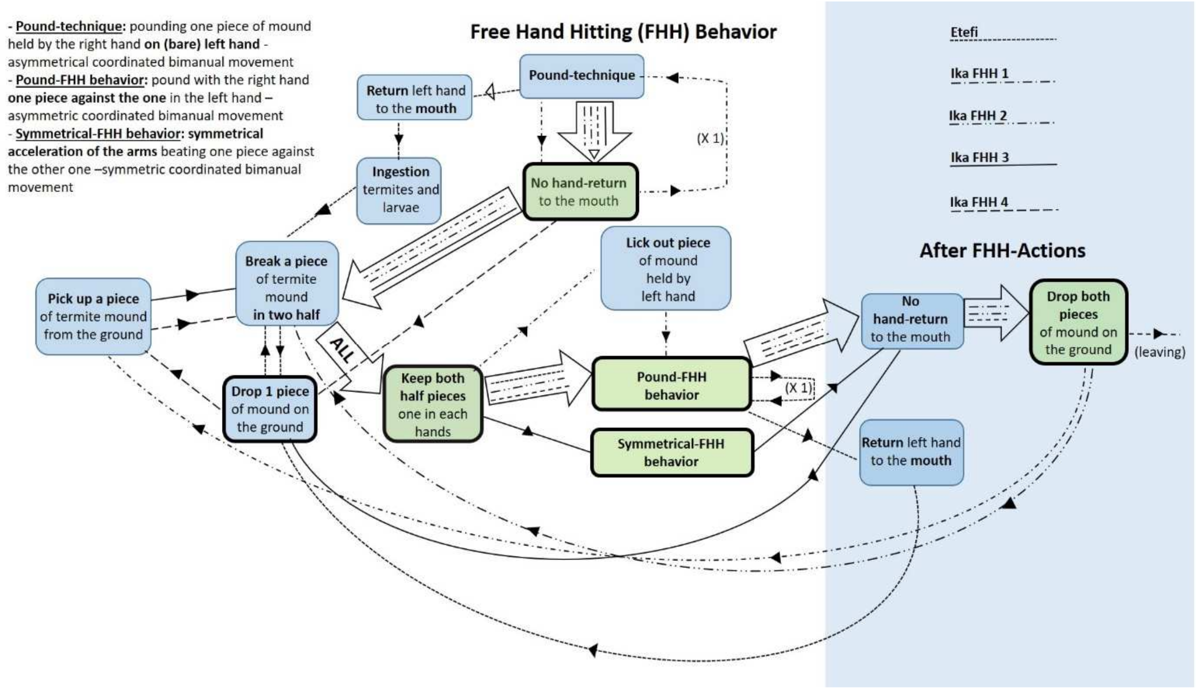

Supplement: Supplementary file 4 — Supplementary Information 1. [file 41598_2022_15542_MOESM4_ESM.pdf]
